# Are Executive Dysfunctions Relevant for the Autism-Specific Cognitive Profile?

**DOI:** 10.3389/fpsyt.2022.886588

**Published:** 2022-07-18

**Authors:** Julia Hemmers, Christopher Baethge, Kai Vogeley, Christine M. Falter-Wagner

**Affiliations:** ^1^Department of Psychiatry, Medical Faculty, Ludwig Maximillians Universitaet (LMU) Munich, Munich, Germany; ^2^Department of Psychiatry and Psychotherapy, Medical Faculty, University Hospital of Cologne, University of Cologne, Cologne, Germany; ^3^Institute of Neurosciences and Medicine—Cognitive Neuroscience, Research Center Juelich, Jülich, Germany; ^4^Department of Psychology, University of Cologne, Cologne, Germany

**Keywords:** autism (ASD), executive function, cognitive profile, theory of mind, weak central coherence

## Abstract

Executive functions (EF) have been shown to be important for the understanding of Autism Spectrum Disorder (ASD), but dysfunctions of EF are not autism-specific. The specific role of EF in ASD, its relationship to core autism characteristics, such as mentalizing, needs to be explored. Medline- and PsychINFO databases were searched for studies published between 1990 and 2020 that included measures of EF in ASD and typically developing control persons (TD) in combination with either Theory of Mind (ToM) or Weak Central Coherence (WCC) tasks. A pre-registered meta-analysis and cross-study regression was performed including a total of 42 studies (ASD *n* = 1,546, TD *n* = 1,206). Results were reported according to PRISMA guidelines. In all cognitive domains, the ASD group showed significantly reduced performance. Importantly, EF subdomains and ToM were not significantly correlated. This finding rules out a significant association between EF subdomains and ToM and questions the relevance of EF dysfunctions for the autism-specific feature of reduced mentalizing.

## Introduction

Autism Spectrum Disorder (ASD) is a neurodevelopmental disorder that primarily comprises symptoms in the social domain with a focus on interaction and communication, as well as restricted repetitive behaviors (1). Aside from the primary symptoms, a disruption of executive functions (EF) has been long proclaimed as a domain-general characteristic of the autistic cognitive profile (2, 3). EF refers to higher cognitive capacities required for goal-directed behavior. The concept of EF consists of multiple subdomains, such as planning, impulse control, set shift, working memory, inhibition, and initiation and monitoring of action (4). Planning involves a dynamic operation in which a sequence of planned actions must be constantly monitored, re-evaluated and updated [e.g., the Tower of London task; (4)]. Set shift, also called mental flexibility, describes shifting to different thoughts or actions according to changes in a situation. Inhibition refers to the ability to inhibit a response and sometimes produce an alternative response. Finally, working memory is needed to maintain and manipulate mental information without external cues. Nevertheless, one should keep in mind that the subdomains are not necessarily clear-cut and that tasks assessing each subdomain may often tap into more than one cognitive domain. For example, to a certain degree, response inhibition additionally utilizes working memory, as it requires holding a rule in one’s mind (5).

Studies regarding atypical EF in individuals with ASD present heterogeneous results. On the one hand, it has been found that individuals with ASD show significantly reduced performance in tasks of EF than typically developed control persons (TD) (6). On the other hand, there are multiple studies that did not elicit differences between groups (7). Yet, there is growing evidence that executive dysfunctions, in general, are found in ASD (6). Nevertheless, it is still unknown whether a particular subset of EF is predominantly impaired in autistic individuals (6) and whether EF bear any relevance for the core symptoms of the autism-specific cognitive profile, such as atypical mentalizing.

The heterogeneity of EF findings across studies makes general statements about the relevance of EF impairments for the autistic neurocognitive profile difficult, and it is unsatisfactory to attribute this heterogeneity to variability in sampling (6). Sources for heterogeneity comprise genetic variability, a distinct gender bias and the presence of various comorbidities. Not only are medical comorbidities more prevalent in ASD persons, psychopathological comorbidities also more commonly occur than in TD persons (8). The most critical aspect about the role of atypical EF in ASD is the lack of specificity. Importantly, EF impairments can be found across neurodevelopmental disorders (9), particularly in attention deficit/hyperactivity disorder (ADHD). ADHD is thought to genetically overlap with ASD (10) but does not share the same phenotype. A recent structural equation modelling study revealed that EF impairments were particularly associated with reported ADHD symptoms in ASD (11).

Additionally, the effects of learning disabilities and ASD on EF are challenging to disentangle. It seems, however, as if a specific profile of executive dysfunction for individuals with ASD exists. In a study investigating EF impairments in two groups of persons with learning disabilities, one with, the other without the comorbidity of ASD, EF impairments were more pronounced in the ASD group (12). While these did not only exist in the ASD group, the sub-domains of planning and working memory effectively differentiated the groups (12).

Furthermore, EF impairments were reported in Tourette’s syndrome, obsessive-compulsive disorder (13), and even in persons with higher Body Mass Index (14); all suggesting a contribution of EF on impulse control. Taken together, the aforementioned analysis of EF in ASD (6) highlights the relevance of EF atypicalities in ASD; yet, an important follow-up question remains: To what extent does atypical EF, which is not autism-specific, bear a role for core symptoms of the autism-specific neurocognitive profile, such as atypical mentalizing? Consequently, EF needs to be investigated in the context of autism-specific characteristics to further evaluate their relevance for etiological and treatment considerations.

For instance, atypical mentalizing specifically prevails in ASD, as described by the reduced Theory of Mind (ToM) account (15). Likewise, a tendency toward Weak Central Coherence [WCC; (16)] is characteristic for the autistic cognitive profile. ToM, or mentalizing, relates to the core clinical symptoms of interaction and communication deficits and reflects the ability to attribute mental states to oneself and others to predict or explain a person’s behavior. Several studies have shown that individuals with ASD show significantly reduced ToM compared to TD individuals [R. (17–20)]. The WCC theory refers to the tendency of autistic individuals to perceive objects, social situations or mental constructs less as a whole and instead focus more specific on details of the holistic structure. Empirically, the WCC was demonstrated by a preference for local over global information processing (21, 22). However, definite conclusions regarding the performance in global processing are difficult to draw since most experiments have mainly studied local processing (16). Thus, evidence for the reduced global processing, in contrast to the preference for local processing, can be considered weak. Nevertheless, WCC has been a prevalent theory studied within the field and is, therefore, considered in the present study. Notably, there are now alternative domain-general accounts for functions categorized under the traditional concept of WCC, including Enhanced Perceptual Functioning (23) and Reduced Generalisation (24).

Yet, none of the previously mentioned theories can primarily explain ASD. Accordingly, it has been proposed to abandon the idea of a single theory explaining ASD (25, 26). Therefore, it is of particular interest to explore the extent to which the unspecific atypical EF in ASD relates to core symptoms of autism-associated domains, ToM and WCC, to understand the relevance of domain-general, and presumably fundamental, EF for the neurocognitive profile of ASD.

The proposal of atypical mentalizing being a consequence of EF dysfunctions has been proposed (4, 27), but few studies have researched the relationship between these domains. Relationships between EF impairments and ToM have likewise been suggested (11). EF deficits in individuals with ASD have, for instance, been associated with reported ADHD-related symptoms in these individuals, whereas ToM impairments were particularly associated with reported ASD symptoms (11). One factor contributing to the development of ToM is the biological maturation enabling children to express their understanding of mental states, which arises from an improvement in EF. Accordingly, the performance on any cognitive task arises from at least two factors: competence (the conceptual understanding required to solve a problem) and performance (other cognitive skills required to access and express understanding; e.g., memory, focussed attention, comprehension, etc.). False belief (FB) understanding, a key aspect of ToM, requires an individual to disengage from a real-world situation to attend to an abstract representation (inhibition/flexibility), to stop a prepotent or habitual response (inhibition; pointing to the actual location of an object), and to hold different and conflicting representations in mind and manipulate this information to come to the correct answer (working memory). Specifically, inhibitory control and working memory seem to be the most relevant skills for ToM development (28, 29). Therefore, deficits in ToM tasks may not be due to pure conceptual limitations but may relate to problems translating conceptual knowledge into successful action. This might result from a failure to flexibly switch between reality and imagination, a failure to inhibit a response, or being unable to withdraw attention from a salient object. Accordingly, there is evidence suggesting a positive correlation between inhibitory control measures and FB tasks (30, 31). Additionally, in a longitudinal study investigating the relationship between EF, WCC, and ToM in children with ASD at two time points, individual differences in early EF skills were shown to influence subsequent performance on ToM tasks, specifically FB tasks (32). By contrast, using a co-twin control design, no association between EF and ToM was found (33).

The evidence regarding the relationship between EF and WCC is mixed. For instance, it was considered that difficulties in global processing might be the result of an inability to switch between the local and global aspects of a stimulus (23, 34). This would suggest a possible relationship between WCC and EF, especially with respect to set shifting. Individuals with ASD might have difficulties on global processing tasks because the tasks require intact EF skills (34). On the other hand, various studies have concluded that there are no developmental links between EF and WCC (35, 36).

Thus, the current pre-registered meta-analysis and cross-study regression aimed to identify whether EF, and if so which particular subdomains, might impact the autistic cognitive profile in terms of autism-specific reduced ToM and WCC.

## Materials and Methods

This systematic review and meta-analysis was pre-registered in the Prospero database (registration number: CRD42019139151).

### Study Selection

Studies were included if published in English or German between January 1990 (by this year, the two concepts of ToM and WCC had been established) and September 2020. Eligible studies had to include participants with a diagnosis of ASD according to DSM-III, DSM-IV, DSM- IV-TR, DSM-5, or ICD-10. The studies had to include assessments of EF measures in combination with either ToM or WCC. Further, we only included studies that provided sufficient data to calculate effect sizes and correlations; no minimum sample size was required.

### Data Collection

We searched Medline (via PubMed) and PsycInfo, applying the following generic literature search:

“Autis*” AND “THEORY OF MIND” AND “EXECUTIVE FUNCTION”

“Autis*” AND “CENTRAL COHERENCE” AND “EXECUTIVE FUNCTION”

To cover a broad range of articles the search terms, *Theory of Mind, Executive Function and Central Coherence* were replaced in the process by numerous synonyms and tests with respect to their domain (Table 1). To reduce a possible selection bias, screening of titles and abstracts was carried out by two evaluators (J.H. & A.B.), both involved in the study but independent of each other.

**TABLE 1:** Search terms.

ASD	Executive function	Theory of mind	Central coherence
-Autis* -Asperger -PDD NOS -ASD -Autism -developmental disorder not otherwise specified -developmental disorder	-Response -Inhibition -Working Memory -Set Shift -Planning Inhibition -Impulse Control -Initiation -Generativity -Tower of London -Tower of Hanoi -Flanker Task -Card Sorting -Wisconsin Card Sorting Task -WCST -Numbers Task -Digit Backwards -Short Term Memory -flexibility	TOM -ToM -mentalizing -Mentalizing -social cognition -socialcognition -RMET -reading the mind in the eyes task -false belief task -false believe task -strange stories -mindreading -SallyAnn Task -Sally-Anne Task -Smarties Task	-local/global Processing -weak central coherence -context -Gestalt -Embedded figures -Detail -Block Design -figure embedding -Navon Figures -Rey’s figure task

PDD NOS, pervasive developmental disorder not otherwise specified; ASD, autism spectrum disorder; WCST, Wisconsin card sorting task; TOM, theory of mind; RMET, reading the mind in the eyes task. *To cover a broader spectrum of search term endings.

The search revealed 71 articles eligible for full-text evaluation. Full-text articles were screened by the aforementioned evaluators. We also screened reference lists of all studies included. Regarding the cognitive construct of EF, not all subdomains were included in the analysis, but only those that were studied in a sufficiently large number of studies (set to a criterion of *n* ≥ 20). The subdomain that was reported the most was cognitive flexibility (*n* = 38 articles), followed by inhibition (*n* = 30), planning (*n* = 22) and working memory (*n* = 20). After filtering the initial 71 articles for the inclusion criteria, 59 articles remained; 17 of these articles were excluded because they only offered limited data. Some articles only reported a mean score, without reporting a minimum or maximum, which did not allow us to calculate percentages (37, 38). Other articles only reported a mean and standard deviation for one cognitive measure (39) or did not report a mean and standard deviation at all (40). Such articles were excluded. A total of 42 articles qualified for the final analysis.

### Data Items and Summary Measures

For each study, the following variables were extracted: title, author, year of publication, IQ (Intelligence Quotient), demographics of participants (including the specifics of the control groups), exclusion criteria of the respective study, as well as the mean, standard deviation, and other measures of dispersion (SE), out of which we calculated standard deviations for our analysis.

### Moderator Analysis

We calculated meta-regressions of each of the individual cognitive domains to analyze associations of differences between ASD and TD groups including average age of study samples and IQ difference between ASD and TD groups (mixed effects regression, method of moments, CMA Version 2).

### Risk of Bias

Two independent raters evaluated each study’s quality using the Newcastle Ottawa Quality Assessment Scale for Case Control Studies (NOS) (41).

### Publication Bias

**FIGURE 1:**
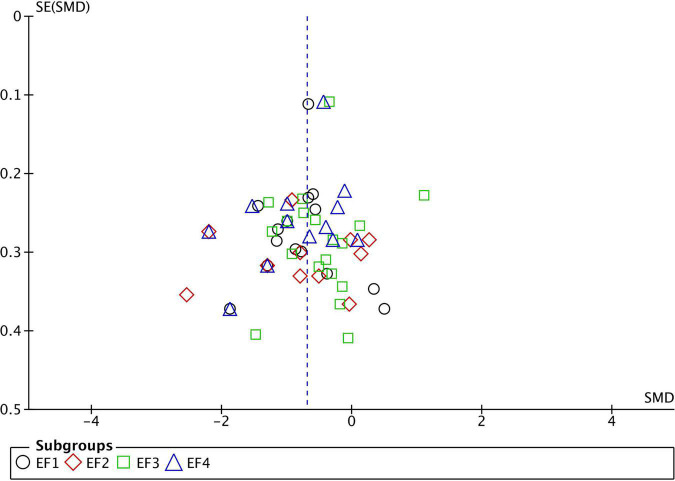
Funnel plot of studies comparing Executive Function performance between ASD and TD. SMD, standard mean difference (Effect Size); SE, standard error; EF1, inhibition; EF2, working memory; EF3, flexibility; EF4, planning. Positive effect sizes indicate superior performance in ASD. The solid vertical line indicates the estimate for the population effect size.

**FIGURE 2:**
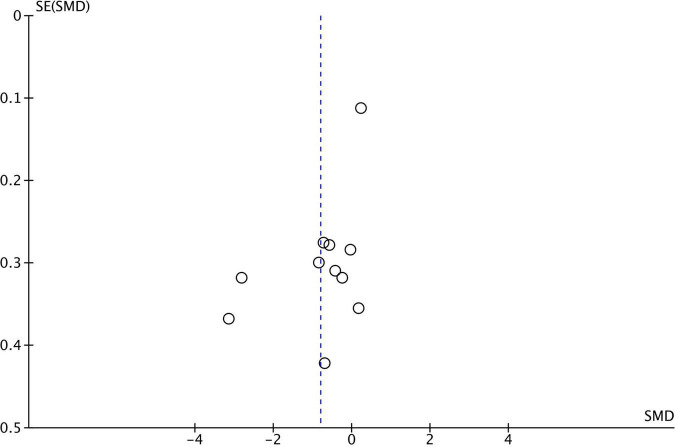
Funnel plot for 11 studies comparing Central Coherence performance between ASD and TD. SMD, standard mean difference (effect size); SE, standard error. Positive effect sizes indicate superior performance in ASD. The solid vertical line indicates the estimate for the population effect size.

**FIGURE 3:**
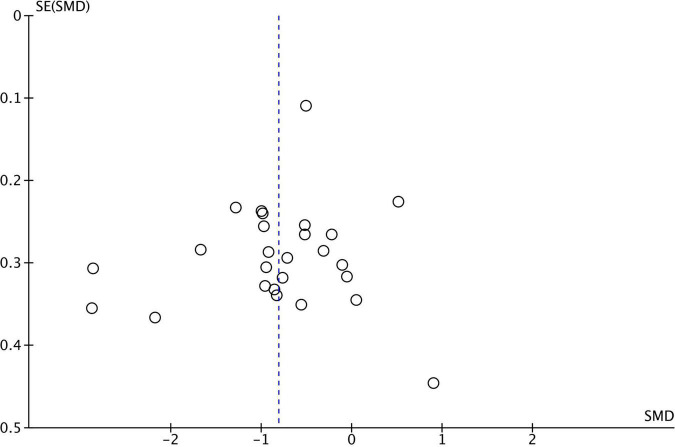
Funnel plot–theory of mind funnel plot for 26 studies comparing theory of mind performance between ASD and TD. SMD, standard mean difference (effect size); SE, standard error. Positive effect sizes indicate superior performance in ASD. The solid vertical line indicates the estimate for the population effect size.

For the analysis of publication bias, we employed funnel plots (Figures 1–3). Funnel plot asymmetry can be indicative of bias that may arise from language, citation, lag time, or publication bias.

### Data Analysis

Data analysis was performed using RevMan 5.3, JMP 15 and CMA (Comprehensive Meta-Analysis Software, v.2.0). All articles included in the final analysis had investigated EF in combination with either ToM or WCC among patients with ASD. Out of the 42 identified studies, nine studies did not have a control group and were subsequently omitted from the calculation of effect sizes, leaving a total of 33 studies.

To quantify the magnitude of the different measures of EF (ToM and WCC), we computed effect sizes (Hedges’ g) of performance in the ASD group relative to the control group, based on means, standard deviations and sample sizes for each measure. For the meta-analysis, a random effects model was used (42). Since the cognitive constructs under study are addressed by a variety of measures, we used a standardized effect estimate across all tests. Effect benchmarks of Hedges’ g can be categorized as the following: g: 0.2–0.5 = “small,” g: 0.5–0.8 = “medium”, g > 0.8 = “large.” For each analysis, we report the sample sizes of ASD and TD groups, the number of studies included, as well as the effect sizes and their 95% confidence intervals. Multiple studies used for the meta-analysis showed effect sizes larger than 1 (with several effect sizes considerably larger than 1; see forest plots), which might indicate possible reporting errors. All authors of the included studies were contacted and asked to confirm the accurate reporting of results. Not all authors replied, but of those who did, all of the data was eventually corrected.

As conservative approach, we conducted an additional separate meta-analysis without studies reporting effect sizes greater than one.

Studies used different tests and scales for measuring the same psychological construct. For example, the concept of inhibition, a subdomain of EF, was measured by the BRIEF (Behavior Rating Inventory of Executive Function), the Luria hand game, the Stroop Inhibition Task, a Card Sorting Task and multiple others (43). The concept of ToM has been measured by tasks like the RMET, the strange stories test and the Sally Ann Test (44). Weak Central Coherence was assessed by tasks such as the embedded figures test, block design tasks and Sentence Completion Tasks (45).

To calculate correlations between different psychological concepts among participants with ASD and to estimate the impact of multiple subdomains of EF on ToM or WCC, we standardized all reported results by transforming them to percentages. In cases where a high test-score indicated bad performance, results were reversed so that, after standardizing, a high percentage indicated a good result.

To choose regression models, normality was analyzed by the Shapiro-Wilk-Test, which showed a normal distribution of almost all measures of cognition. Therefore, we calculated Pearson’s *r* with 95% confidence intervals between the cognitive constructs of EF subdomains, ToM and WCC, using JMP 15 as a meta-analytical tool. Since the concept of WCC could only be correlated with very few studies, this variable was omitted from further calculations.

A total of 11 studies had to be excluded for the correlation analysis mainly due to missing data, leaving a total of 31 studies that were eligible for this part of the analysis.

**FIGURE 4:**
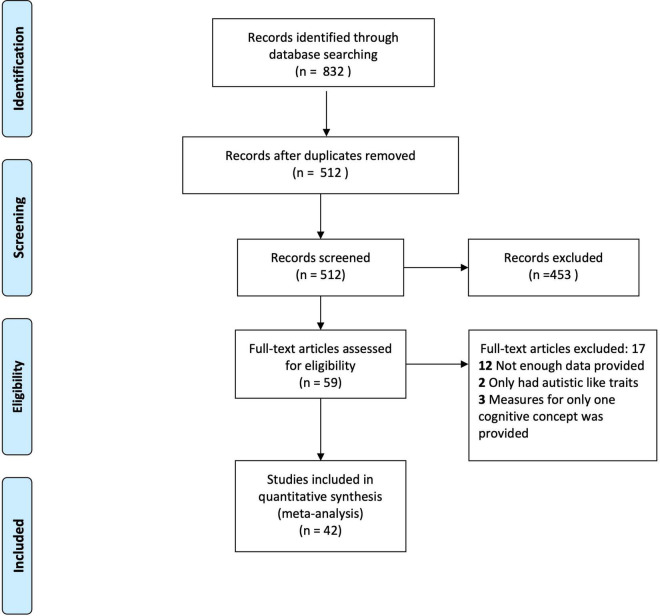
PRISMA flowchart.

We followed the Preferred Reporting Items for Systematic Reviews and Meta-Analysis (PRISMA) (46). For further detail see PRISMA Flowchart (Figure 4).

## Results

### Retrieved Studies

Data came from a total of 42 studies used for the final analysis. The literature search yielded data from 1,546 participants with ASD (grand mean age = 13.36, SD = 8.37, mean age range: 4.95–35.46, grand median age = 10.05 years), that were compared to 1,206 typically developing individuals (grand mean age = 13.17, SD = 11.85, mean age range: 4.1–38.3 years, grand median age = 8.94 years). Sample sizes ranged from a minimum of 10 participants to a maximum of 181.

Of the 42 studies observed, 36 had measured IQ. The grand mean IQ for the ASD group was *M* = 101.82, SD = 10.76 and that of the TD group was *M* = 104.03, SD = 21.63. There was no significant effect of IQ between the two groups *t*_(33)_ = 0.739, *p* = 0.536.

### Meta-Analysis: Cognitive Constructs

**FIGURE 5:**
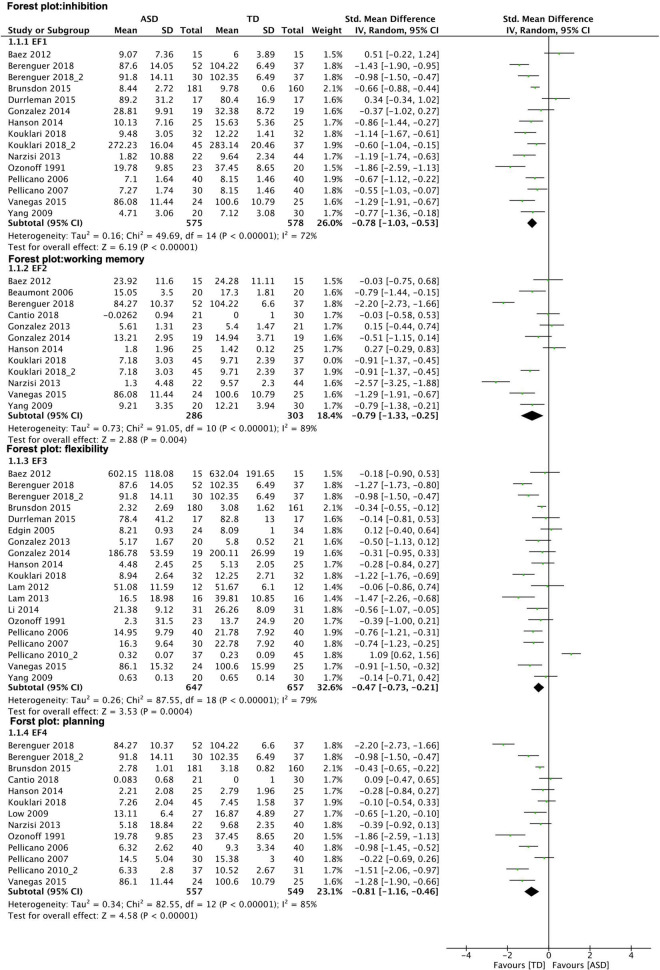
Forest plot–executive function: subgroup analysis of EF domains. Results show medium effect size measures for all EF subtypes observed. Graphical explanations: horizontal lines present 95% confidence Interval of the effect sizes for each study; green dot: hedges‘g; diamond shape: overall effect size.

The different domains of EF that were analyzed showed moderate to large effect sizes: inhibition (EF1), *k* = 15, g = -0.78, 95% CI [-1.03, -0.53], I^2^ = 72% ASD *n* = 575 TD *n* = 578; working memory (EF2), k = 11, g = -0.79, 95% CI [-1.33, -0.25], I^2^ = 89%, ASD *n* = 286, TD *n* = 303; flexibility (EF3), k = 19, g = -0.47, 95% CI [-0,73, -0.21], I^2^ = 79%, ASD *n* = 647, TD *n* = 657; planning (EF4), *k* = 13, *g* = -0.81, 95% CI [-1.16, -0.46], I^2^ = 85%, ASD *n* = 557, TD *n* = 549. See results in Figure 5.

Participants with ASD (*n* = 873) performed poorer compared to TD participants (*n* = 870) in ToM tasks, with a large effect size (*k* = 24, *g* = -0.81, 95% CI [-1.10, -0.52], I^2^ = 87%).

Regarding WCC, participants with ASD (n = 403) showed lower test scores relative to TD participants (*n* = 413), with a large effect size (*k* = 9, *g* = -0.80, 95% CI [-1.43, -0.17], I^2^ = 93%).

Since some studies showed effect sizes larger than 1, a complementary analysis was performed with all studies reporting effect sizes less than 1.

The different domains of EF that were analyzed showed small to moderate effect sizes: the matter of inhibition (*k* = 10, *g* = -0.53, 95% CI [-0.76, -0.29], I^2^ = 56%), with a total of *n* = 422 participants with ASD and *n* = 420 TD participants; working memory (k = 8, g = -0.34, 95% CI [-0.68, -0.01], I^2^ = 64%, ASD *n* = 188, TD *n* = 197); flexibility (*k* = 15, *g* = -0.44, 95% CI [0.59, -0.28], I^2^ = 27%) with *n* = 510 participants with ASD and *n* = 527 TD participants planning (k = 9, g = -0.44, 95% CI [-0.66, 0.22], I^2^ = 53%, ASD *n* = 421, TD *n* = 436).

Participants with ASD performed poorer (*n* = 682) compared to TD participants (*n* = 696) in ToM tasks, with a moderate effect size (*k* = 19, *g* = -0.51, 95% CI [-0.71, -0.31], I^2^ = 66%).

Regarding WCC, participants with ASD (*n* = 326) showed lower test scores relative to TD participants (*n* = 342), with a small effect size (*k* = 7, g = -0.31, 95% CI [-0.64, 0.02], I^2^ = 71%).

### Correlation Analysis

In general, the correlations between the EF subdomains (working memory, inhibition, planning, and flexibility) and ToM can be categorized as small for the ASD and the TD group. None of the correlations showed significance (*p* > 0.05); therefore, only the minimum and maximum correlations are reported. Correlations ranged from a maximum of r(6) = 0.143, *p* = 0.353 for working memory in the TD group to a minimum of r(22) = 0.0040, *p* = 0.7667 between planning and ToM in the ASD group.

### Moderator Analysis

In meta-regressions, we found no association between effect sizes and IQ difference between ASD and TD groups. When looking at WCC, a significant effect for age was observed (slope = 0.22, *p* = 0.031, df = 10). The difference in WCC measures between the ASD and TD group became weaker with increasing age of participants. For ToM, no such effect was observed.

### Risk of Bias

For assessing the risk of bias for individual studies, the NOS was evaluated. It showed an average overall score of 5.21 points. The NOS scale ranges from zero to nine points. Regarding analysis of studies that were included, a minimum of two points and a maximum of eight points were observed. Based on the results of this analysis, the quality of studies that were analyzed can be considered between of “fair” and “good” quality. After filtering all studies by quality, a further meta-analysis was performed that only included studies that were rated as “good” (at least six points). The studies that qualified for analysis can be drawn from Table 2.

**TABLE 2:** Included studies.

			ASD	Group		TD	Group		Cognitive	Construct				
Source and publication	Diagnostic criteria	NOS scale	*N*	*M* age (SD)	*M* IQ (SD)	*N*	*M* Age (SD)	*M* IQ (SD)	Inhibition	Working memory	Flexibility	Planning	ToM	CC
Baez et al. (58)	DSM IV	6 (good)	15	35.46 (11.86)	-	15	35.7 (11.52)	-	X	X	X			
Beaumont and Sofronoff (59)	DSM IV	7 (good)	25	9.52 (0.88)	114 (12.26)		9.52 (0.88)	119.69 (10.49)		X			x	
Berenguer et al. (60)	ADI-R	7 (good)	52	8.59 (1.38)	101.42(12.65)	37	8.54 (1.26)	102.11 (8.91)	X	X	X	X	X	
Berenguer et al. (61)	ADI-R	7 (good)	30	8.39(1.3)	100.37 (12.4)	37	8.54 (1.2)	102.11 (8.9)	X	X	X	X	X	
Beversdorf et al. (40)	ADI-R	6 (good)	10	30.8 (9.3)	109.7 (16.2)	13	30.6 (12)	117.3 (11.2)					X	
Brunsdon et al. (62)	ADOS ADI-R	6 (good)	181	13.49 (0.69)	94.07 (16.91)	160	12.79 (1.1)	102 (12.79)	X		X	X	X	X
Cantio et al. (63)	DSM IV	8 (good)	21	10.7 (1.5)	105.48 (15.95)	30	10.96 (1.26)	109.47 (18.58)		X		X	X	X
Durrleman and Franck (64)	DSM IV	6 (good)	17	9 (2)	-	17	7.2 (xx)	-	X		X		X	
Edgin and Pennington (65)	ADI-R	6 (good)	24	11.46 (2.32)	104.4 (20.4)	34	12.4 (2.52)	108.72 (13.04)			X			X
Gonzalez-Gadea et al. (66)	DSM IV	7 (good)	23	33 (9.8)	-	21	38.3 (14.2)	-		X	X		X	
Gonzalez-Gadea et al. (37)	DSM IV	7 (good)	19	11.89 (2.64)	101.93 (11.96)	19	10.89 (2.3)	100.59 (12.2)	X	X	X		X	
Hanson and Atance (67)	DSM IV	5 (fair)	25	5.2 (1.49)	85.71 (21)	25	4.1 (0.93)	109.12 (8)	X	X	X	X	X	
Jones Catherine et al. (44)	ICD -10	2 (poor)	100	15.6(0.6)	84.31 (18.03)	-	-	-	X	X	X	x	X	
Joseph and Tager-Flusberg (51)	DSM IV	2 (poor)	31	8.9 (2.5)	88 (22.8)	-	-	-	X	X		X		
Kouklari et al. (38)	DSM IV DSM V	6 (good)	45	9.07 (1.42)	97.05 (12.13)	37	9.03 (1.17)	102.11 (14.3)	X	X		X	X	
Kouklari et al. (68)	DSM IV	6 (good)	32	10.34 (1.29)	100.69 (12.85)	32	10 (1.35)	114.81 (9.98)	X	X	X		X	
Kimhi (20)	DSM IV	7 (good)	29	4.95 (11.06)	103.52(17.21)	30	4.6 (10.97)	107.6 (14.13)				X	X	
Lam (69)	-	4 (fair)	16	8.9 (1.41)	108.44 (12.76)	16	8.42 (2.07)	109.75 (12.58)			X			X
Lam and Yeung (70)	-	5 (fair)	12	6.11 (8.23)	70.17 (3.51)	12	5.64 (1)	77.91 (3.04)			X			X
Low et al. (71)	DSM IV	6 (good)	27	8.26 (2.17)	-	27	6.6 (1.31)	-				X	X	X
Lai et al. (72)	-	6 (good)	64	28.15 (6.1)	114.15 (11.15)	64	27.55 (7.7)	113.5 (14.9)	X				X	
Lukito et al. (11)	ADI-R	2 (poor)	100	-	84.3(18)	-	-	-	X	X	X	X	X	
Le Sourn-Bissaoui et al. (73)	DSM IV	6 (good)	10	16.1 (3.5)	101.8(17.06)	10	15.9 (3.6)	98.4 (10.9)						
Lind et al. (74)	DSM IV	6 (good)	20	8.67 (1.37)	105.65(16.34)	20	8.31 (0.91)	109.05 (8.68)						X
Livingston et al. (75)	-	6 (good)	136	13.28 (0.39)	-	136	13.28 (0.39)	-	X	x	X	X	X	
Loth et al. (45)	DSM IV	2 (poor)	21	16.8 (6.2)	89.9(23.4)	10	6.3 (1.1)	-						X
Lehnhardt et al. (76)	ICD-10	6 (good)	39	31.1 (8.9)	127.9(16.2)	39	31.2 (8.1)	133.3 (11.6)			X		x	
Miranda et al. (77)	-	7 (good)	52	8.59 (1.38)	101.42 (12.65)	39	8.46 (1.27)	102.21 (8.7)	x				x	
Montgomery et al. (78)	-	2 (poor)	25	18.2 (1.38)	114(11.1)	-	-	-	X					
Narzisi et al. (79)	DSM IV	7 (good)	22	9.77 (3.65)	99.09(14.23)	40	9.77 (3.65)		X	X		X	X	
Ozonoff et al. (3)	DSM III	6 (good)	23	12.05 (3.19)	89.52(15.17)	20	12.39 (3.04)	91.3 (18.75)	x		X	X		
Pellicano (80)	-	7 (good)	30	5.63 (0.97)	100.03 (10.55)	40	5.47 (0.95)	103.25 (9.92)	X		X	X	X	
Pellicano (32)	DSM IV	4 (poor)	45	5.42 (0.87)	113.27(13.93)	45	5.43 (1.05)	115.61 (16.42)	X		X	X	X	X
Pellicano (36)	DSM IV	6 (good)	45	5.6 (0.87)	113.27 (13.93)	-	-	-			X	X	X	X
Pellicano et al. (81)	DSM IV	6 (good)	40	5.59 (0.83)	101.15 (11.04)	40	5.47 (0.95)	103.25 (9.91)	X		X	X	X	X
Schuwerk et al. (82)	ICD-10	7 (good)	14	8 (1.8)	-	21	7.2 (1.4)	-					X	
Stichter et al. (83)	ADI-R ADOS	4 (poor)	20	8.77 (1.3)	99.3(15.18)	-	-	-	X	X	X	X	X	
Vanmarcke et al. (84)	DSM IV	5 (fair)	24	20.63(0.38)	107.63(8.7)	24	20.83 (0.41)	108.9 (6.05)	X	X	X	X	X	
Vanegas and Davidson (85)	-	7 (good)	24	9.7 (1.35)	100.72 (14.32)	25	8.86 (1.09)	110.12 (14.59)	X	X	X	X		X
Williams et al. (86)	-	5 (fair)	21	10.6 (2.01)	110.19(16.35)	21	10.59 (1.31)	107.48 (13.23)			X		X	
Yang et al. (87)	DSM IV	6 (good)	20	15.5 (8.1)	96.68 (24.63)	30	8 (3.1)	118.23 (12.06)	X	X	X		X	
Zelazo et al. (88)	DSM III–R	2 (poor)	22	13.88 (4.75)	42.59 (13.32)	-	-	-	X		X		X	

*This Table presents Demographics of the samples observed and the cognitive Constructs that were measured for each study. ASD, autism spectrum disorder; TD, typically developing; NOS, Newcastle Ottawa scale; ToM, theory of mind; CC, central coherence.*

The different domains of EF that were analyzed showed large to moderate effect sizes: for the matter of inhibition (*k* = 14, *g* = -0.77, 95% CI [-1.04, -0.51], I^2^ = 74%), with a total of *n* = 550 participants with ASD and *n* = 553 TD participants; working memory (*k* = 11, *g* = -0.9, 95% CI [-1.38, -0.42], I^2^ = 87%, ASD *n* = 306, TD *n* = 315); flexibility (k = 16, g = -0.55, 95% CI [-0.75, -0.35], I^2^ = 59%) with *n* = 582 participants with ASD and *n* = 584 TD participants planning (k = 11, g = -0.79, 95% CI [-1.17, -0.42], I^2^ = 86%, ASD *n* = 495, TD *n* = 493).

Participants with ASD showed reduced performance (*n* = 766) compared to TD participants (*n* = 769) in ToM tasks, with a moderate effect size (k = 20, g = -0.78, 95% CI [-1.10, -0.46], I^2^ = 88%).

Regarding WCC, participants with ASD (*n* = 338) showed lower test scores relative to TD participants (*n* = 354), with a moderate effect size (*k* = 6, *g* = -0.65, 95% CI [-1.29, -0.02], I^2^ = 92%).

### Publication Bias

Funnel plots were generated for each analyzed cognitive construct. All constructs included data of at least 10 studies. Funnel plots did not indicate a publication bias as demonstrated in Figures 1–3.

## Discussion

This pre-registered meta-analysis aimed to investigate the relevance of dysfunctional EF including subdomains on the core symptomatic autism-specific cognitive profile. In comparison to TD control persons, participants with ASD showed significantly reduced performance in all three cognitive domains: EF, ToM, and WCC. The largest group differences were found in ToM, in the EF subdomain of planning and WCC. As shown in the moderator analysis, it is possible that findings regarding WCC are due to age, having a modulatory effect on the findings. This remains a limitation of the present study, especially when considering that we investigated a developmental disorder. The observed age span ranged from 4 to 38 years, which is difficult to compare due to noted differences across the lifespan. Only a minority of five studies showed an average age greater than 18 years (N ASD = 123; N TD = 98). To account for this limitation, future research should include matched control groups to better compare the differences in ToM, EF, and WCC between ASD and control individuals. In addition, given the heterogeneity of ASD, some aspects of cognition are more affected in different age groups. Interestingly, cognitive abilities, such as visual and verbal memory, persist across adulthood in ASD, while other cognitive abilities become less apparent in old age (47).

With respect to EF, we found reduced performance in persons with ASD with large to moderate effect sizes for all studied subdomains in the following descending order: planning, working memory, inhibition, and flexibility. These results are in accordance with previous findings of atypical EF in ASD (6, 48). Age-related differences in EF were also observed in children and adolescents with ASD (49, 50). van den Bergh et al. (49) found that inhibition problems were reported less for the older children and adolescents. However, the opposite effect was observed for planning, such that older children and adolescents had more difficulties with planning. Therefore, these findings highlight the heterogeneity of the disorder, thereby emphasizing the need to focus on individual differences when studying EF in ASD.

Concerning the main question of the current study, the regression analyses showed no significant association between any of the EF subdomains and ToM, neither for the ASD nor the TD group. To our knowledge, no previous studies have looked at the relationship between these two cognitive domains employing meta-analytical tools; although previous studies have suggested that the impairment in ToM, which is regularly observed in ASD, might be understood as a consequence of EF dysfunctions (4, 27). The lack of a significant association between EF and ToM rather points at independent dysfunctions. These findings can be taken to suggest that atypical mentalizing should rather be regarded as distinctly autism-specific (51), given the lack of association with domain-general EF atypicalities and despite having been reported for other developmental disorders and conditions [e.g., (9, 13, 14)]. Notably, data used for the regression analysis came from a relatively small group; therefore, results should be cautiously interpreted and rather understood as an explorative approach toward the hypothesis of independent dysfunctions of EF and ToM. Nonetheless, our results are not in accordance with the suggestion of atypical ToM as a result of atypical EF (4, 27). Due to limitations in the number of studies, a correlation analysis for the EF subdomains and WCC was not appropriate; yet, WCC is reported herein according to pre-registration practices. The association between EF and WCC will need to be re-evaluated in future studies once sufficient evidence has been accumulated.

As a common limitation in ASD research, the included samples were very heterogeneous in multiple aspects, such as sample size and inclusion criteria. For instance, some studies allowed for comorbidities or measured the severity of symptoms whereas others did not. Therefore, all participants that were included showed highly different degrees of impairment, limiting comparability but, at the same time, reflecting the nature of the spectrum. Likewise, studies were heterogeneous in terms of paradigms and methodology. For instance, among the four subdomains of EF that were included, there were at least 16 different tasks reported. Moreover, the tests were often adapted for specific samples (e.g., a version for children), or the tests were shortened due to limited attention of participants. Furthermore, the array of tests was not individually checked for validity in the present study. This suggests that a more critical evaluation of task comparability would be useful for future investigations. The clinical and methodological heterogeneity is reflective of the research activities in the whole field, and while we accounted for this by applying random effects models, it calls for cautious interpretation.

Reduced categorization of the ToM tasks remains a further limitation of the data collection. ToM tasks of higher and lower order were not separately assessed but all summarized and considered ToM tasks in the wide sense. Since the cognitive requirements greatly differ depending on the given task in a particular study, the findings may be affected by concentration and cognitive ability. Furthermore, the EF subdomains were not discretely defined, which may also have additionally contributed to the variability.

Some included studies reported unusually high effect sizes. A previous study that had looked at subgroups of EFs also noted relatively high effect sizes. They noticed that high effect sizes were present in studies that had used self-report data or questionnaire data (6). Hence, we inspected our data set for measurements that were used in studies with unusually high effect sizes. This observation was not present in the current data set. Of the 10 studies that had reported effect sizes greater than 1, only two used questionnaires. With exception of WCC, our findings achieved in this conservative analysis remained largely unchanged after excluding studies with particularly high effect sizes.

The independence of EF and ToM renders EF as an unspecific symptom of developmental and neurological conditions (52, 53), and is suggestive of a genetic overlap between developmental disorders (10, 54). Regarding the practical relevance of our findings, children who are being trained in EF skills cannot readily be expected to show a generalization of improvement in mentalizing skills given the independence of functions.

An important factor to consider is the ecological validity of the assessments used to measure cognitive ability and executive functioning in ASD. Ultimately, the goal of research is to apply research findings into practice in a real-world setting. While executive tests are widely used as a measure of EF, studies have found that not all neuropsychological tests have strong ecological validity (55). Therefore, more focus should be placed on developing ecologically valid assessments to measure cognition and executive functioning in ASD. One study introduced the contextual assessment of social skills (CASS), which assesses the conversation ability of participants as a more ecologically valid alternative (56). Results showed strong internal validity of the CASS, and to some extent, external validity of the CASS as a measure of social cognition (56). Since ASD is characterized by atypical communicative and interactive behavior, assessments such as the CASS could be a more ecologically valid measure of social cognition among individuals ASD. Another possibility would be to use multiple types of presentation formats and tasks during assessments to better parse between problems with application versus ability (57).

## Conclusion

This meta-analysis and regression showed that there is an overall atypical pattern of performance in ASD for all classical cognitive constructs: EF, ToM, and WCC. Although all EF subdomains were significantly different in the ASD group with a moderate effect size, EF did not show any significant association with the autism-specific domain of mentalizing.

## Data Availability Statement

The original contributions presented in the study are included in the article/supplementary material, further inquiries can be directed to the corresponding authors.

## Author Contributions

CB supported regarding statistics. KV supported in terms of content. All authors contributed to the article and approved the submitted version.

## Conflict of Interest

The authors declare that the research was conducted in the absence of any commercial or financial relationships that could be construed as a potential conflict of interest.

## Publisher’s Note

All claims expressed in this article are solely those of the authors and do not necessarily represent those of their affiliated organizations, or those of the publisher, the editors and the reviewers. Any product that may be evaluated in this article, or claim that may be made by its manufacturer, is not guaranteed or endorsed by the publisher.
